# Analysis of effectiveness of a surgical treatment algorithm for basal cell carcinoma*

**DOI:** 10.1590/abd1806-4841.20165919

**Published:** 2016

**Authors:** Flávio Barbosa Luz, Camila Ferron, Gilberto Perez Cardoso

**Affiliations:** 1 Universidade Federal Fluminense (UFF) – Niterói (RJ), Brazil; 2 Private clinic – Rio de Janeiro (RJ), Brazil

**Keywords:** Mohs surgery, Carcinoma, basal cell, Treatment outcome, Recurrence

## Abstract

**BACKGROUND:**

Surgical excision is the treatment of choice for basal cell carcinoma and micrographic surgery considered the gold standard, however not yet used routinely worldwide available, as in Brazil. Considering this, a previously developed treatment guideline, which the majority of tumors were treated by conventional technique (not micrographic) was tested.

**OBJECTIVE:**

To establish the recurrence rate of basal cell carcinomas treated according to this guideline.

**METHOD:**

Between May 2001 and July 2012, 919 basal cell carcinoma lesions in 410 patients were treated according to the proposed guideline. Patients were followed-up and reviewed between September 2013 and February 2014 for clinical, dermatoscopic and histopathologic detection of possible recurrences.

**RESULTS:**

After application of exclusion criteria, 520 lesions were studied, with 88.3% primary and 11.7% recurrent tumors. Histological pattern was indolent in 85.5%, 48.6% were located in high risk areas and 70% small tumors. Only 7.3% were treated by Mohs micrographic surgery. The recurrence rate, in an average follow-up period of 4.37 years, was 1.3% for primary and 1.63% for recurrent tumors. Study limitations: unicenter study, with all patients operated on by the same surgeon.

**CONCLUSION:**

The treatment guideline utilized seems a helpful guide for surgical treatment of basal cell carcinoma, especially if micrographic surgery is not available.

## INTRODUCTION

Incidence of basal cell carcinoma (BCC) is increasing. In the USA, more than 3.5 million new cases of nonmelanoma skin cancer were estimated for 2014, and it was also noted that the number of women under 40 diagnosed with BCC more than doubled in the last 30 years.^1^

Although less than 50% of the Brazilian population is Caucasian, nonmelanoma skin cancer is also prevalent in Brazil, representing 25% of all malignant tumors.^2^ For 2014 it was estimated 182,130 new cases of nonmelanoma skin cancer, and BCC corresponded to 70% of these diagnoses. ^3^

Surgical resection is the treatment of choice for BCC.^4,5^ Currently, in the USA, Mohs micrographic surgery (MMS) is indicated for all recurrent BCCs, except for superficial ones in low risk areas. For primary tumors, it is indicated for all aggressive tumors (except smaller than 0.5cm in low risk areas); for all nodular tumors in high and moderate risk areas and for those larger than 2cm in low risk areas; and for all superficial tumors in high risk areas and for those largest than 0.6cm in moderate risk areas.^6^

An increase of 400% in the use of MMS in the US from 1995 to 2009 was reported, and one in four skin cancers are treated this way.^6^ In Brazil, MMS was introduced in the 1980's and, although widely accepted, its application is still limited, mainly due to the small number of specialized services.^7,8^

**Figure 1:**
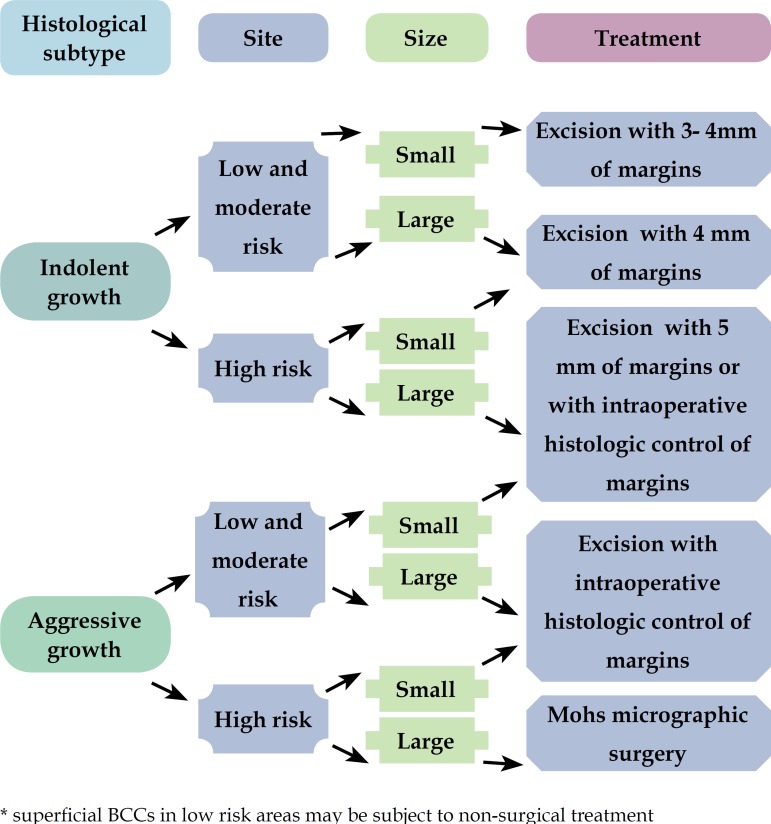
Algorithm for surgical treatment of primary BCC

**Figure 2:**
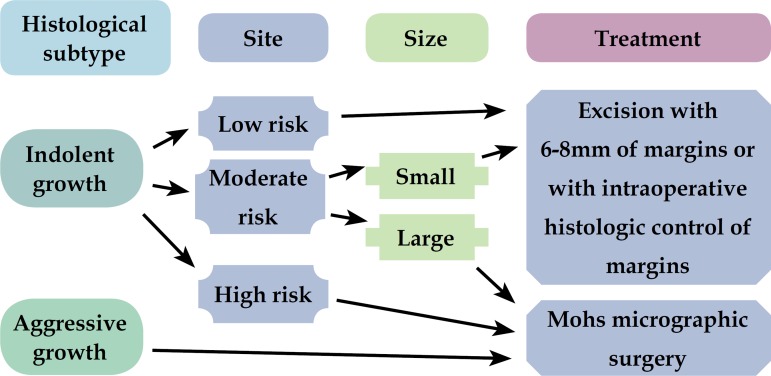
Algorithm for surgical treatment of recurrent BCC

Because MMS is not yet widely and routinely used in many countries, the authors formulated in 2001 an algorithm to guide the surgical treatment of BCC, especially for places where there is still no broad access to micrographic techniques (Figures 1 and 2). ^9^

The objective of this study was to evaluate the cure rate of BCCs patients treated surgically according to this algorithm.^9^ Although non-surgical techniques are accepted for some cases of BCCs, they were not covered in this study.

## METHODS

Patients diagnosed with BCC treated in a private treatment center skin cancer were analyzed. The study involved patients treated between May 2001 and July 2012.

**Figure 3:**
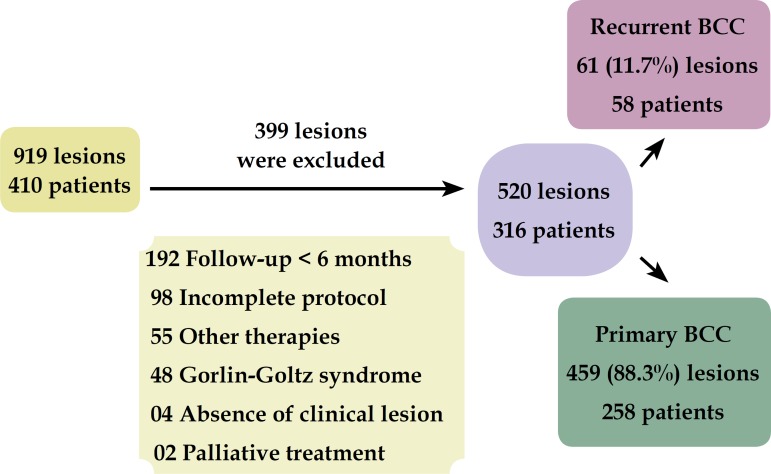
Exclusion criteria

The study included: 1) lesions with histologic diagnosis of BCC treated surgically according to the proposed algorithm; and 2) patients who agreed to the proposed treatment. Exclusion criteria were: 1) patients with follow-up less than 6 months after treatment; 2) patients undergoing treatments other than surgery; 3) cases with no clinically visible lesion (previously treated by other doctors and referred to center after incomplete resection); 4) patients with Goltz-Gorlin syndrome; and 5) locally invasive lesions undergone palliative treatment (Figure 3).

The project was approved by the Ethics Review Board of the Universidade Federal Fluminense (document number: 155.435).

After initial classification of tumors in primary or recurrent, the pattern of histological growth was verified, according to previous biopsy. Presence of perineural invasion, metatypical sclerodermiform, infiltrative and micronodular subtypes were classified as aggressive growth; and nodular and superficial subtypes, as indolent growth, according to the Crowson classification.^10^

Thereafter, the location of the tumor was classified as high, moderate or low risk, according to the Huang and Boyce classification.^11^

The tumor size classification takes into account its location. Thus, we considered large lesions those greater than 1cm, located in high risk areas, those greater than 2cm in moderate risk areas and those greater than 4cm located in low risk areas.

After this stratification, the tumors were treated according to the algorithm (Figures 1 and 2). Some primary BCCs, superficial, located in low risk areas, were treated by non-surgical methods.^12^

All patients were treated by the same surgeon (FBL).

The demarcation of tumor margins was made after clinical skin degreasing with 70% alcohol, using surgical focus and maintaining the skin slightly stretched. From 2006, the polarized light dermoscopy (Dermalite II Pro^®^) started to be used to assist such delimitation.

In case of involvement of any of the margins in the histological analysis, new resections were made to confirm the total removal of the tumor.

From September 2013 to February 2014, patients were invited to attend the review consultation (with neutral observer), during which clinical signs and dermatoscopic relapse were sought. For patients who were unable to attend this consultation, telephone contact was made and data of the last visit made by the same surgeon were considered.

## RESULTS

We evaluated 919 lesions in 410 patients. Of these, 301 met the exclusion criteria and 98 had incomplete registry data, preventing its inclusion. Thus, 521 lesions in 316 patients were effectively studied, 459 of these were primary tumors and 61 were recurrent tumors.

The general characteristics of the sample are shown in table 1.

**Table 1:** Characteristics of the sample

	Total	Primary BCC	Recurrent BCC
Nr. of lesions	520	459 (88.3%)	61 (11.7%)
Nr. of patients	316	258 (81.6%)	58 (18.4%)
Sex			
Women		171 (54.2%)	139 (53.8%)
Men		145 (45.8%)	119 (46.1%)
Mean age	68.83 years	68.82 years	67.77 years
	(de 30 a 98)		
Women		70.16 years	67.55 years
Men		67.36 years	68.25 years
Mean follow-up	4.37years(±2.52)	4.42 years	3.98 years
	6 m/12y5m	6 m/12y5m	6 m/10y6m
Minimum/Maximum			
< 5 years	331 (63.6%)	288 (62.7%)	44 (72.1%)
≥ 5 years	189 (36.4%)	171 (373%)	17 (27.9%)

### Histologic subtype of tumors

The nodular subtype was the most frequent, both in the group of primary and recurrent tumors (64.8% and 40.9%, respectively) (Table 2). When tumors were classified according to histologic growth pattern, the majority of primary and recurrent tumors were indolent (87.9% and 59.1%, respectively).

**Table 2:** Histological subtype

Histological subtype	Primary BCC* N: 281	Recurrent BCC** N: 44	Histological subtype	Primary BCC* N: 281
Sclerodermiform	10 (3.5%)	4 (9.1%)	Indolent growth	252 (89.7%)
Infiltrative	3 (1.1%)	6 ( 13.6%)	Aggressive growth	29 (10.3%)
Metatypical	1 (0.3%)	2 (4.5%)		
Mixed	15 (5.3%)	5 (11.4%)		
Nodular	182 (64.8%)	18 (40.9%)		
Micronodular	-	1 (2.3%)		
Superficial	63 (22.5%)	8 (18.2%)		
Pigmented	5 (1.8%)	-		
Ulcerated	2 (0.7%)	-		

* Not reported: 178

** Not reported: 17

### Tumor site

The vast majority of primary (63.6%) and recurrent (85.2%) tumors were located in the head and neck. In the group of primary tumors, trunk was the most frequent isolated place (26.44%). In the group of recurrent, nose was the most commonly affected (36%), followed by the forehead (16.4%) (Table 3).

**Table 3:** Tumor site

Site	Primary BCC	Recurrent BCC	Site	Primary BCC	Recurrent BCC
Nose	100 (21.8%)	22 (36%)	High risk	218 (47.5%)	35 (57.4%)
Perioral	40 (8.7%)	2 (3.3%)			
Temporal	27 (5.9%)	1 (1.7%)			
Periocular	26 (5.6%)	2 (3.3%)			
Ears and periauricular	22 (4.8%)	8 (13.1%)			
Jaw 5 (1.1%)	1 (1.6%)				
Forehead	32 (7%)	10 (16.4%)	Moderate risk	77 (16.8%)	19 (29.5%)
Scalp	5 (1.1%)	2 (3.3%)			
Cheek	24 (5.2%)	4 (6.5%)			
Neck	11 (2.4%)	0			
Upper limbs	14 (3%)	2 (3.3%)	Low risk	164 (35.7%)	8 (13.1%)
Lower limbs	32 (7%)	2 (3.3%)			
Trunk	121 (26.4%)	5 (8.2%)			

In relation to risk areas, 47.5% of primary tumors and 57.4% of recurrent were located in high risk areas.

### Tumor size

Considering the tumor size, 88.6% of primary and 73.2% of recurrent tumors had maximum diameter of 2cm (Table 4).

**Table 4:** Tumor size

Size	Primary BCC* N: 280	Recurrent BCC** N: 41
≤ 2cm	248 (88.6%)	30 (73.2%)
> 2cm	32 (11.4%)	11 (26.8%)
Small	213 (76%)	12 (29.3%)
Large	67 (24%)	29 (70.7%)

* Not reported: 179

** Not reported: 20

Most primary (76%) and almost a third (29.3%) of recurrent tumors were small according to their location.

### Treatment

The vast majority of primary and recurrent tumors were treated by conventional surgery (CS) (94.5% and 75.4%, respectively), with 62% of primary and 74% of recurrent tumors subjected to intraoperative histological analysis of their margins (Table 5).

**Table 5:** Treatment

Treatment	Primary BCC*	Recurrent BCC**
Conventional surgery	94.5% (434)*	75.4% (46)**
Postoperative histological analysis of margins (paraffin)	25.8% (112)	13% (6)
Postoperative histological analysis of margins (frozen section)	62% (269)	74% (34)
Mohs micrographic	5.5% (25)	24.6% (15)

* Not reported: 53

** Not reported: 6

The rate of incomplete excision, among tumors treated by conventional surgery with intraoperative histological analysis of margins, was 4.1% (18 lesions) for primary and 4.3% (two lesions) for recurrent tumors. Among primary tumors, the lateral margin was involved in 13 cases, the deep margin in two, and both margins, lateral and deep, in three cases. Among the recurrent tumors, one case presented lateral margin and the other presented deep margin affected. All were called for subsequent surgery and all underwent additional resection until confirmation of complete tumor excision.

Among the 466 tumors with available information, the surgical repair occurred in 56.9% (265) for simple flap; 19.5% (91) by direct closure; 14% (65) by complex flap; 8.5% (40) graft; and 1% (5) by second intention.

### Follow-up and recurrence of the algorithm

The mean follow-up was 4.37 years (minimum 6 months and maximum 12 years and 5 months), with a mean of 4.42 years among primary tumors and 3.98 years among recurrent tumors.

**Figure 4:**
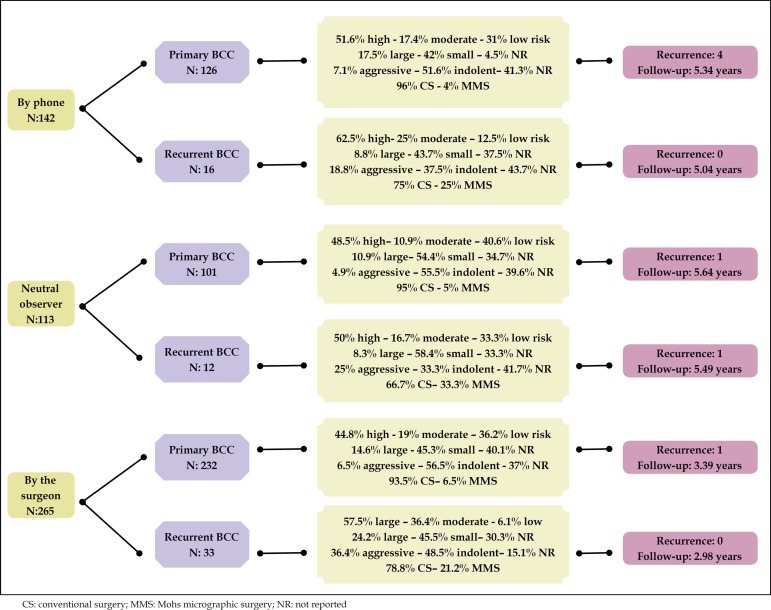
Characteristics of the sample according to the reviewer

The review consultation was performed with 21.7% (113) patients by a neutral observer; 51% (265) by the same surgeon; and via telephone contact by neutral observer in 27.3% (142) of the cases. In the latter group, 28.2% (40) of the cases mentioned follow-up by another doctor (Figure 4).

The overall relapse rate was 1.34%, 1.3% (6) between primary and 1.63% (1) between recurrent tumors.

In two of the cases of recurrence, the lesions were located in the nose, and in two others, the lesions were clinically nodular (Table 6).

**Table 6:** Characteristics of the cases of recurrence treated according to the algorithm

Patient	Tumor status	Histological / clinical types	Site	Size	Treatment
1	Primary	Nodular/Nodular	Scalp	Small	CS with frozen section– 4mm margins
2	Primary	Sclerodermiform / Nodular	Nose	Large	CS with frozen section – 5mm margins
3	Recurrent	NR/NR	Nose	NR	MMS
4	Primary	NR/Nodular	Nose	Large	CS with frozen section – 4mm margins
5	Primary	NR/Nodular	Nose	NR	MMS
6	Primary	NR/ Sclerodermiform	Nose	Small	CS with frozen section – 3mm margins
7	Primary	Nodular/Nodular	Temporal	Small	CS with frozen section – 3mm margins

CS: conventional surgery; NR: not reported; MMS: Mohs micrographic surgery

Most recurrences occurred in the nose and in tumors clinically classified as nodular.

With the exception of lesion 3, which has not yet been re-operated, other cases were reoperated according to the recurrent tumors algorithm and, so far, they didn't present a new relapse (Table 6 and Figure 2).

## DISCUSSION

The discreet prevalence in women in this study was similar to previous reports, although high prevalence in men has also has been reported.^13-16^ Mean age was 65 years (minimum of 30 and maximum of 98 years) and was similar to previous reports.^13,15,16^

Similar to other reports, the nodular histologic subtype was the most frequent. There are reports showing the nose as the most affected site, but in this study, the trunk was the most common site among primary tumors (26.4%), followed by the nose (21.8%), which was the most frequent among the recurrent tumors (36%).^14,17,18,19^ Similar to other studies, approximately half of the primary and recurrent tumors were small (54% and 49.2%, respectively).^13,18^

Analyzing the cases according to the criteria adopted and tested in the algorithm (histologic growth pattern, risk areas and size according to location), 10.3% of primary tumors and 40.9% of recurrent tumors were aggressive; 47.5% of primary and 57.4% of recurrent tumors were located in high risk areas; and 24% of primary and 70.7% of the recurrent tumors were large.

Only 5.5% of primary and 24.6% of recurrent tumors were treated by MMS. If we applied the US indications of MMS^6^ to our sample, instead of the proposed algorithm, more than 70% of the treated cases would have indication for MMS.

More than half (63.1% - 303 of 480) of tumors treated by conventional surgery underwent intraoperative histological analysis of margins, and incomplete excision rate was 4.1% (20 of 480). Bariani *et al.* ^14^ obtained 8% of positive margins, excising well defined BCCs, smaller than 20 mm, with surgical margins of 3 mm, and poorly defined BCCs, larger than 20mm, with 5mm margins. Nagore *et al.* obtained 24% of positive margins excising BCCs with margins of 2 to 3 mm.^20^ Sherry *et al.* obtained incomplete excision rate of 3.2% excising primary BCCs with minimum margins of 3mm.^16^ Pichardo-Velazquez *et al.* obtained incomplete excision rate of 28.5%, excising high risk BCCs with surgical margins of 5mm. ^21^

The mean follow-up was 4.37 years (36.3% of the lesions were followed for more than 5 years), which is close to what is considered ideal by Gulleth *et al.*^22^

The overall recurrence rate of this study was 1.34%; 1.30% among primary tumors and 1.64% among recurrent tumors. Among the six cases of recurrence, which occurred in primary tumors, five were treated by conventional surgery, with complete removal of the tumor confirmed by the intraoperative histological analysis of margins, and one was treated by MMS. The only tumor that recurred in the group of previously treated lesions was located in high risk area and treated by MMS.

Although Fleischer *et al.* claim that the surgeon's experience does not affect the probability of incomplete resection, as all lesions were treated by the same surgeon, it would be interesting that the algorithm was tested in other centers.^23^

There are reports of recurrence in 5 years of 1% for primary BCCs treated by MMS and of 10.1% for those treated by conventional surgery; and of 5.6% for recurrent tumors treated by MMS and of 17.4% the ones treated by conventional surgery.^24^ Mosterd *et al.*, in a prospective randomized study, obtained recurrence of 4.1% for primary BCCs treated by conventional surgery and of 2.5% for those treated by MMS, in a mean follow-up of 5 years.^25^ For recurrent tumors, they obtained recurrence of 12.1% for conventional surgery and of 2.4% for MMS. Cigma *et al.*, excising BCCs with margins between 3 and 10 mm, according to their location, obtained recurrence of 2.6%.^26^ Wetzig *et al.* reported recurrence in 5 years of 0.5% for primary BCCs and of 2.9% for the excised recurrent BCCs with histologic control in paraffin.^18^ Rowe *et al.* reported recurrence in 5 years of 1% for primary BCCs and of 5.6% for recurrent tumors treated by micrographic techniques.^27,28^ Some studies involving periocular BCCs, excised with intraoperative histological analysis of margins, reported relapse of 2.15% and 9.7% for primary tumors and of 4.4% and 14.2% for recurrent tumors.^29-31^

Although the results of this study are similar to those obtained with MMS, we do not consider the sacrifice of healthy skin that, although not tested, is greater than with micrographic techniques. This observation has been demonstrated by Muller *et al.* that, in a randomized study, obtained a mean surgical defect of 111.6 mm² for small nodular BCCs treated by MMS versus 187.7mm² for conventional surgery. ^32^

Among the excluded cases, there was a recurrence in a patient with Gorlin-Goltz syndrome, treated by conventional surgery, one in a patient submitted to MMS for palliation and one in a patient treated by photodynamic therapy.

## CONCLUSION

Complete excision is the key to surgical treatment of BCC. Accordingly, MMS has its indications well established in the literature and is the treatment of choice for most cases. However, considering the cure rate obtained in these studies, to sites where MMS is not yet widely available, the proposed algorithm can be a useful guide to direct the surgical treatment of basal cell carcinoma.
